# Factors contributing to chronic ankle instability in parcel delivery workers based on machine learning techniques

**DOI:** 10.1186/s12911-025-02919-7

**Published:** 2025-02-13

**Authors:** Ui-jae Hwang, Oh-yun Kwon, Jun-hee Kim, Gyeong-tae Gwak

**Affiliations:** 1 College of Health Science, Laboratory of KEMA AI Research (KAIR), Yonsei University, 234 Maeji-ri, Heungeop-Myeon, Wonju, Kangwon-Do, 220–710 South Korea; 2 College of Health Science, Laboratory of Kinetic Ergocise Based on Movement Analysis, Yonsei University, 234 Maeji-ri, Heungeop-Myeon, Wonju, Kangwon-Do, 220–710 South Korea

**Keywords:** Ankle, Chronic ankle instability, Machine learning, Physical therapy/Rehabilitation, Injury prevention

## Abstract

**Background:**

Ankle injuries in parcel delivery workers (PDWs) are most often caused by trips. Ankle sprains have high recurrence rates and are associated with chronic ankle instability (CAI). This study aimed to develop, determine, and compare the predictive performance of statistical machine learning models to classify PDWs with and without CAI using postural control, ankle range of motion, ankle joint muscle strength, and anatomical deformity variables.

**Methods:**

244 PDWs who had worked in parcel delivery for more than 6 months were screened for eligibility. Thirteen predictors were included in the study: 12 numeric (age, body mass index, work duration, the number of balance retrials eyes-closed single-limb stance, Y-balance test, ankle dorsiflexion range of motion, lunge angle, strength ratio of the evertor in plantar flexion and neutral position to the invertor, ankle dorsiflexor strength, navicular drop, and resting calcaneal stance position) and one categorical (success of the eyes-closed single-limb stance). Five machine learning algorithms, including LASSO logistic regression, Extreme Gradient boosting machine, support vector machine, Naïve Bayes machine, and random forest–were trained.

**Results:**

The support vector machine and random forest models confirmed good predictive performance in the training and test datasets, respectively, for PDWs. For the Shapley Additive Explanations, among the five machine learning models, the variables entered into three or more models were low ankle dorsiflexion range of motion, low lunge angle, high body mass index, old age, a high number of balance retrials of the eyes-closed single-limb stance, and low strength ratio of the evertor in the neutral position to the invertor.

**Conclusion:**

Our approach produced machine learning models to classify PDWs with and without CAI and confirmed good predictive performance in PDWs.

## Background

Since the pandemic, logistics and retail industries have grown rapidly with the development of online shopping and e-commerce in the global economy, thereby increasing the employment of parcel delivery workers (PDWs) [1–4]. PDWs handle various weights, sizes, and shapes of parcels from the warehouse to the doorsteps of clients. A general parcel delivery system includes a combination of driving and walking [5]. A study showed that PDWs walk an average of 8 km during delivery work, which includes repeatedly getting in and out of the truck, walking, and running with parcels, though the round distance to a parcel distribution center was excluded in the study [6].

Lateral ankle sprains are the second most common work-related musculoskeletal disorder [7]. Studies have shown that approximately 77% of ankle sprains involve the lateral ligaments, making lateral ankle sprains particularly prevalent [8]. The ankle is the most frequent site of injury (23%) in PDWs, and ankle injuries are most often caused by trips [9]. Ankle injuries occur in unpredictable, uncontrolled, and varied outdoor environments during delivery [10]. Occupational risk factors for ankle injuries include damaged and uneven paving and obstacles on the walking surface, stairways and steps, lighting conditions, and unsafe working behaviors such as rushing, taking hazardous shortcuts, entering dangerous areas, and reading addresses while walking during delivery work [10]. However, because ankle sprains have a high rate of recurrence, which is related to the development of chronic ankle instability (CAI) [11, 12], it is necessary to consider not only occupational risk but also functional insufficiency factors, including sensory-perceptual and motor-behavioral impairments, to prevent and manage recurrent ankle sprains.

Ankle sprains result in an average of 20 lost working days [7], and recurrences due to CAI can result in more lost working days. Therefore, accurate classification of PDWs with and without CAI is required, followed by the management and recovery of PDWs with CAI by improving the factors contributing to CAI. Functional insufficiencies contributing to CAI after lateral ankle sprain are caused by alterations in the motor component of sensorimotor control, particularly impaired postural control [13, 14], limited ankle ROM [15, 16], diminished ankle eversion strength [17, 18], anatomical deformities [19], and changes in motor control of the muscles proximal to the injured ankle [20, 21]. However, because the factors contributing to CAI vary with mechanical insufficiency and occur independently or in combination, the factors contributing to CAI remain controversial for researchers and clinicians alike [18, 22].

Most classification modeling research on CAI has used either linear or logistic regression as a statistical method to identify key contributing factors according to whether the outcomes are continuous or binary [18]. However, linear and logistic regression methods are disadvantageous when the relationships between the outcome (or logit of the outcome) and the contributing factors are nonlinear. Nevertheless, machine learning (ML) is increasingly being used to classify models. Because ML has the advantage of a suite of algorithms that could model highly nonlinear and linear relationships, it has been recommended to achieve higher accuracy compared with traditional statistical methods. The five machine learning models (LASSO logistic regression, Extreme Gradient boosting machine, support vector machine, Naïve Bayes machine, and random forest) were selected based on their proven effectiveness in medical classification tasks and their complementary approaches to classification problems. LASSO logistic regression provides interpretable linear relationships, support vector machine excels at handling non-linear boundaries, random forest offers robust performance with feature importance rankings, Extreme Gradient boosting provides high accuracy through sequential learning, and Naïve Bayes offers probabilistic classification with computational efficiency [23]. Thus, the primary purpose of the present study was to develop, determine, and compare the predictive performance of statistical ML models to classify PDWs with and without CAI using postural control, ankle range of motion (ROM), ankle joint muscle strength, and anatomical deformity. The secondary purpose of the present study was to identify the contributing factors that have a significant impact on CAI and explain a model that could help prevent and manage CAI in PDWs by improving these factors.

## Methods

### Participants

Overall, 244 PDWs who had worked in parcel delivery for more than 6 months were screened for eligibility. PDW’s data generated from musculoskeletal screening tests for preventing industrial accident were used by visiting a musculoskeletal health care center in delivery company from August 2021 to March 2022. Informed consent for the present study was waived by Institutional review board before the queries and analyses because it was an analysis based on data already obtained by the parcel delivery company. Of the 244 PDWs included in the study, participants were classified into either the control group (n = 184) or the CAI group (n = 60, 24.6%) based on their ankle sprain history and stability status. Control group included participants with no history of ankle sprains and those who had experienced ankle sprains but reported no episodes of ‘giving way’ or feeling of ankle instability. Among those with a history of ankle sprain(s), participants were classified into the CAI group if they met al.l of the following criteria: (1) at least one ankle sprain resulting in lateral ankle pain and impaired physical activity, (2) at least one episode of ‘giving way’ or feeling of ankle instability, and (3) a score of ≥ 4 points on the Ankle Instability Instrument [15], confirming recurrent instability. While the presence of giving way episodes was confirmed through the Ankle Instability Instrument, the specific frequency could not be quantified as the instrument only assesses the occurrence (yes/no) rather than the number of episodes. Participants were excluded if they had a recent history (< 6 months) of lower-extremity surgery, a diagnosis of ankle osteoarthritis, or a history of ankle surgery involving intra-articular fixation. To analyze the relationship between approximately 14 variables, a sample size of at least 140 participants was required, following the 1:10 rule of thumb [24].

### Outcome measures

#### Postural control: Y-balance test and one leg standing with eye closed

Participants performed six practice trials and two measurement trials for the Y-balance test (YBT) and stood with both hands placed on their chests. The patients were instructed to reach three 3 lines as far as possible in the anterior, posteromedial, and posterolateral directions [25]. To perform the eyes-closed single-limb stance (ECSLS) test, participants were required to stand on one leg and remain as still as possible with their hands on their hips and eyes closed for 20 s [26]. If participants could not maintain this position without falling, they could place their non-stance limb on the ground, regain balance as quickly as possible, and continue the balance trial (three trials, each separated by a 30 s break) [26].

#### Ankle range of motions: ankle dorsiflexion range of motion and lunge angle

Ankle dorsiflexion ROM in a straight knee while sitting was measured using a smart phone (SM-G960N, SAMSUNG, Suwon, Korea) installed Clinometer application (Plaincode, Stephanskirchen, Germany) on the lateral side of the foot with the knee joint in terminal extension, with the participant seated on the examination table and the distal half of the lower leg extending past the edge of the Table [27]. The participants were asked to relax as the tester passively dorsiflexed the talocrural joint until a restriction was reached, as indicated by the firm endpoint. The dorsiflexion angles were recorded. An Clinometer application was used to measure the tibial angle in the lunged position [28]. The smart phone was placed 15 cm below the tibial tuberosity. While lunging, the participant aligned the second toe and heel in a straight line on the ground. The participants lunged, trying to touch a vertical line on the wall with their knees while holding heel contact with the ground [28]. The lunge angle was measured at the end position of the tibial advancement.

#### Ankle joint muscles strength: evertor, invertor, and dorsiflexor

In measuring the ankle muscle strength, the strap of the Smart KEMA strength sensor (KOREATECH, Inc, Seoul, Korea) was applied to the distal end of the metatarsal to allow half of the foot to be off the table, and the opposite side of the sensor was fixed to the absorber on the floor using a belt [29]. Ankle muscle strength (kgf) was collected over 5 s, and the average of the middle 3 s was processed. The neutral and plantar flexion positions were set at 0° and 50°, respectively, to measure ankle evertor strength. The participants were asked to lie on their opposite sides to that of the leg to be measured and bend their hip and knee at 90° [29]. The investigator manually fixed the distal tibia of the participant to prevent external tibial torsion and instructed the participant to evert the ankle while maintaining slight toe flexion with maximum force [29]. For the measurement of ankle inverter strength, the participants were asked to lie on the same side as that of the leg to be measured, and they were asked to bend their hip and knee at 90° while maintaining the neutral ankle position. The investigator manually fixed the distal tibia of the participant to prevent internal tibial torsion and instructed them to invert their ankle with maximum force. Moreover, the participants were asked to sit on a table to measure ankle dorsiflexor strength; bend their hips and knee to 90° and hold their ankles in a neutral position; and dorsiflex while maintaining slight toe flexion with maximum force.

#### Anatomical deformities: navicular drop and resting calcaneal stance position

The participants stood on the floor to measure the navicular drop, and the navicular tuberosity was marked. The foot was everted and inverted until the talus was centrally positioned to determine the subtalar neutral position [30]. The distance between the navicular tuberosity and the floor was measured in the subtalar neutral and relaxed positions. In measuring the resting calcaneal stance position (RCSP), participants were asked to lie prone on a bed horizontal to the ground with their feet over the edge of the bed. Participants drew a bisection line, based on three dots on the upper, middle, and lower parts of the calcaneus [31]. The RCSP was measured when the participants stood with their feet first width apart. The angle between the bisection line of the calcaneus and the line perpendicular to the ground was defined as the RCSP [31].

### Screening tests protocol

Participants completed a brief health history and patient-reported Ankle Instability Instrument. Subsequently, PDWs with CAI were measured on the affected side, and those without CAI were measured on the dominant side for the YBT, one leg standing with eyes closed, ankle dorsiflexion range motion, lunge angle, navicular drop, RCSP, and ankle joint muscle strength. All the tests were performed barefooted. The order of measurement of ankle joint muscle strength was randomized, and the participants rested for 3 min between each measurement of ankle muscle strength. In the event of bilateral CAI, the side with greater ankle instability was used for data analysis.

### Data source and collection

Patient characteristics, including age, body mass index (BMI), and work duration during parcel delivery, were collected. The average of the two measured values was applied to the anatomical deformities, postural control, ankle ROM, and ankle joint muscle strength. For the ECLS, the success of the ECSLS and the number of balance retrials due to balancing failure were collected. For the YBT, the composite score was calculated as the sum of the highest reaches in each of the three directions divided by three times the leg length [25]. For ankle muscle strength, the strength ratios of the evertor to invertor and dorsiflexor muscles were collected. The navicular drop was defined as the difference between the neutral and relaxed subtalar positions [30]. For RCSP, calcaneal eversion and inversion were collected as positive and negative values, respectively.

### Machine learning modeling

ML analysis was performed using the Orange data mining software (Orange 3.3.0, Ljubljana, Slovenia) and Python (Version 3.6.15; Python Software Foundation).

#### Pre-processing and missing data handling

Thirteen predictors were included in the study: 12 numeric (age, BMI, work duration, the number of balance retrials of ECSLS, YBT, ankle dorsiflexion ROM, lunge angle, strength ratio of the evertor in plantar flexion and neutral position to the invertor, ankle dorsiflexor strength, navicular drop, and RCSP) and one categorical (success of the ECSLS). Exploratory data analysis was used to confirm missing data, and imputation was performed to remove instances with unknown values. The distribution of each variable was confirmed as a boxplot to remove outliers because they influenced the accuracy of the learning model.

#### Machine learning algorithm

We split the complete data (*n* = 244) into a training set (80%, *n* = 196) for model development and a test set (20%, *n* = 48) to externally validate the prediction performance. Five ML algorithms, including LASSO logistic regression, Extreme Gradient boosting machine, support vector machine, Naïve Bayes machine, and random forest–were trained using 10-fold cross-validation. All ML algorithms have one or more parameters whose values are used to control the learning process to optimize the predictive accuracy of the model for hyperparameter tuning.

#### Model validation

The primary measure of model performance was the area under the curve (AUC) for the training and test datasets (average over classes). The secondary measures of model performance were the classification precision, recall, and the F1 score (average over classes). The F1 score is the harmonic mean of precision and recall, providing a single score that balances both measures. The score ranges from 0 to 1, where 1 represents perfect precision and recall. It is calculated as: F1 = 2 × (Precision × Recall)/(Precision + Recall). The predicting performance was categorized as excellent (≥ 0.9), good (0.8–0.9), fair (0.7–0.8), and poor (< 0.7) by the AUC value [32].

For each clinical outcome, the feature permutation importance was calculated to identify important factors in the trained model. The importance of feature permutations was applied to the training data to compute the contribution of each feature to the prediction (based on the AUC) by measuring the increase in the prediction error of the model. Additionally, a Shapley additive explanation summary plot was made to display the importance and direction of each predictive variable, which their position on the y-axis was sorted by relative importance, with the most important predictors at the top. For each predictive variable, the position of each point (red indicates higher values or the presence of binary factors) on the x-axis expresses the contribution of individual participants to the overall Shapley additive explanation value, with high positive contributions on the far right. Shapley Additive Explanations is a game theoretic approach that explains the output of any machine learning model by calculating the contribution of each feature to the prediction. Shapley Additive Explanations values represent a feature’s importance for a particular prediction, with positive values indicating the feature increased the probability of the predicted outcome, and negative values indicating it decreased the probability.

## Results

### PDWs characteristics

Table 1 shows the means and standard deviations of all variables. Overall, 244 PDWs were included in the ML analysis, of whom 24.6% (*n* = 60) met the criteria for CAI. All PDWs who participated in this study were male. The means and standard deviations of the Ankle Instability Instrument score were 5.9 ± 1.2 and 2.3 ± 1.5 in PDWs with and without CAI, respectively. The proportions of the side with CAI were 18.3%, 31.7%, and 50.0% on the right (*n* = 11), left (*n* = 19), and both sides (*n* = 30; the side with the greater Ankle Instability Instrument: right = 12 and left = 18), respectively. The proportions of the dominant side in PDWs without CAI were 97.8% and 2.2% on the right (*n* = 180) and left (*n* = 4) sides, respectively.

**Table 1:** Mean (standard deviation) of baseline characteristics in PDWs with and without CAI

	with CAI (*n* = 60)			Without CAI (*n* = 184)			*p*
Age (yr)	38.20	±	6.14	37.23	±	8.82	0.431
Work duration (day)	394.59	±	156.60	407.80	±	271.17	0.721
BMI (kg/m^2^)	25.33	±	4.11	23.44	±	3.46	0.001
Evertor strength in neutral position (kgf)	21.63	±	7.02	25.65	±	8.55	0.000
Evertor strength in plantar flexion (kgf)	15.12	±	4.10	17.77	±	6.50	0.000
Invertor strength(kgf)	25.55	±	7.22	29.62	±	8.45	0.000
Ratio of evertor in neutral/invertor (%)	89.63	±	36.81	88.81	±	25.97	0.873
Ratio of evertor in plantar flexion/invertor (%)	64.19	±	29.57	61.23	±	18.66	0.469
Ankle dorsiflexor strength (kgf)	38.05	±	11.75	41.29	±	13.64	0.077
Ankle dorsiflexion ROM (°)	2.11	±	5.19	5.00	±	4.00	0.000
Lunge angle (°)	38.61	±	8.83	45.05	±	30.63	0.011
Navicular drop (cm)	0.68	±	0.51	0.65	±	0.32	0.539
RCSP (°)	2.37	±	3.47	1.45	±	3.65	0.086
YBT (%)	93.41	±	13.25	112.83	±	13.38	0.018
Success of ECSLS (0/1)	48/12 (0/1)			112/72 (0/1)			0.007
Number of balance retrials of ECSLS (trial)	2.14	±	1.81	1.90	±	2.42	0.426

### Predictive models of machine learning

**Fig. 1:**
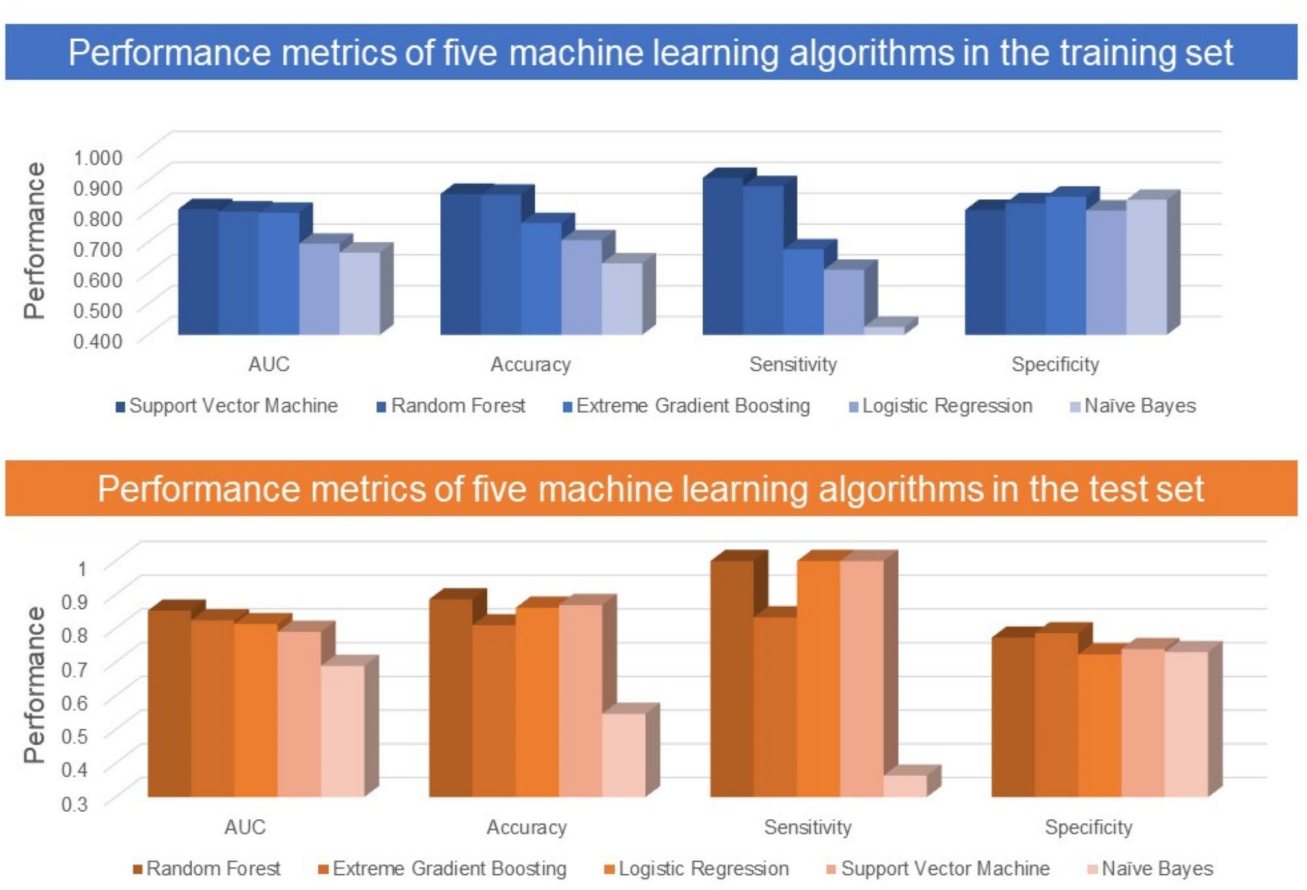
Performance metrics of five machine learning algorithms in the training and test set

**Fig. 2:**
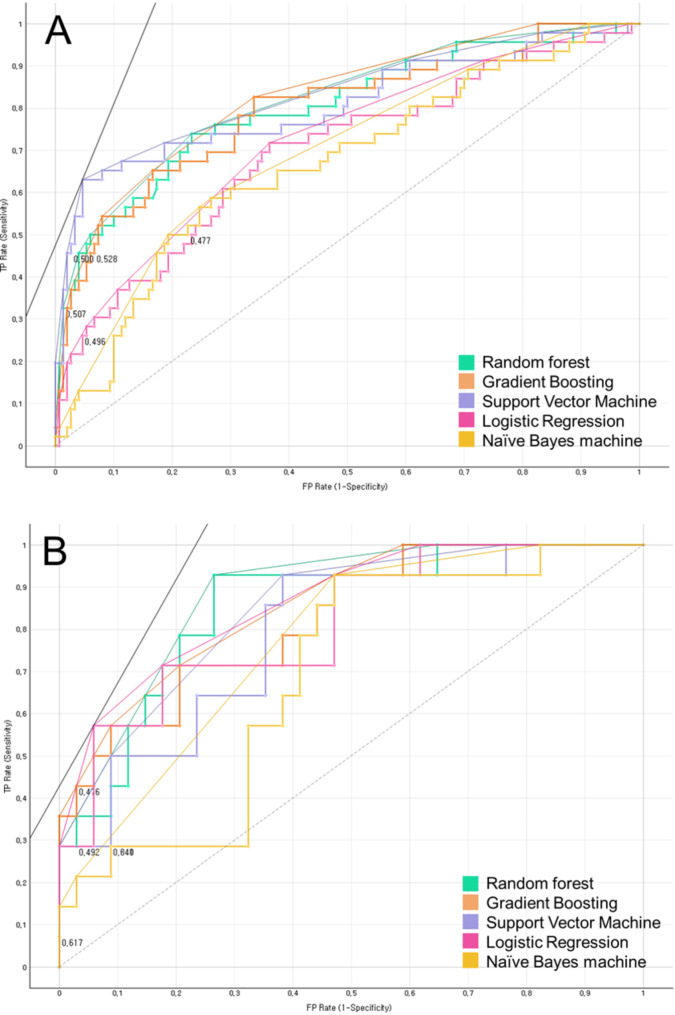
Receiver operating characteristic (ROC) curves of five machine learning algorithms **A**: in the training set, **B**: in the test set

Table 2; Fig. 1 present the performances of the five ML models for predicting CAI during model training and testing, and Table 3 shows the most important predictors. Five ML models during the training model classified PDWs with and without CAI, performed in the order of high AUC, support vector machine [AUC, 0.807 (good)], random forest [AUC, 0.800 (good)], Extreme Gradient boosting [AUC, 0.796 (fair)], LASSO logistic regression [AUC, 0.696 (poor)], and Naïve Bayes machine [AUC, 0.667 (poor)] (Table 2; Fig. 2). During the test model classification of PDWs with and without CAI, five ML prediction models were performed in the following order: high AUC, random forest [AUC, 0.853 (good)], Extreme Gradient boosting [AUC, 0.824 (good)], LASSO logistic regression [AUC, 0.813 (good)], support vector machine [AUC, 0.790 (fair)], and Naïve Bayes machine [AUC, 0.689 (poor)] (Table 2; Fig. 2).

**Table 2:** Performance metrics of five machine learning algorithms in the training and test set

Performance metrics of five machine learning algorithms in the training set				
Performing model	AUC	F1	Precision	Recall
Support vector machine	0.807	0.763	0.830	0.811
Random forest	0.800	0.800	0.840	0.832
Extreme gradient boosting	0.796	0.808	0.808	0.821
Logistic regression	0.696	0.748	0.758	0.786
Naïve Bayes	0.667	0.732	0.741	0.724
Performance metrics of five machine learning algorithms in the test set				
Performing model	AUC	F1	Precision	Recall
Random forest	0.853	0.747	0.839	0.792
Extreme gradient boosting	0.824	0.761	0.800	0.792
Logistic regression	0.813	0.634	0.804	0.729
Support vector machine	0.790	0.675	0.815	0.75
Naïve Bayes	0.689	0.632	0.623	0.646

**Table 3:** Most important predictors in five machine learning models

Performing model	Most important predictors	
	Feature permutation importance	Shapley Additive Explanations
Random Forest	Ankle dorsiflexion ROM, strength ratio of evertor in a neutral position to invertor, age, lunge angle, BMI, YBT	Low ankle dorsiflexion ROM, high BMI, low lunge angle, low strength ratio of evertor in a neutral position to invertor, old age, high RCSP
Extreme Gradient Boosting	Age, ankle dorsiflexion ROM, strength ratio of evertor in plantar flexion to invertor, lunge angle, BMI, strength ratio of evertor in a neutral position to invertor	Low ankle dorsiflexion ROM, old age, low strength ratio of evertor in plantar flexion to invertor, low strength ratio of evertor in a neutral position to invertor, low lunge angle, high BMI
Logistic Regression	Ankle dorsiflexion ROM, success of the ECSLS, RCSP, ankle dorsiflexor strength, lunge angle, a number of balance retrials of the ECSLS	Fail of the ECSLS, low ankle dorsiflexion ROM, high RCSP, low ankle dorsiflexor strength, low lunge angle, a high number of balance retrials of the ECSLS
Support Vector Machine	Success of the ECSLS, ankle dorsiflexion ROM, RCSP, the number of balance retrials of the ECSLS, age, strength ratio of evertor in a neutral position to invertor	Fail of the ECSLS, low ankle dorsiflexion ROM, a high number of balance retrials of the ECSLS, low strength ratio of evertor in a neutral position to invertor, low lunge angle, high RCSP
Naïve Bayes	Ankle dorsiflexion ROM, RCSP, age, strength ratio of evertor in plantar flexion to invertor, BMI, ankle dorsiflexor strength	Low ankle dorsiflexion ROM, old age, high BMI, long work duration, a high number of balance retrials of the ECSLS, high ankle dorsiflexor strength

**Fig. 3:**
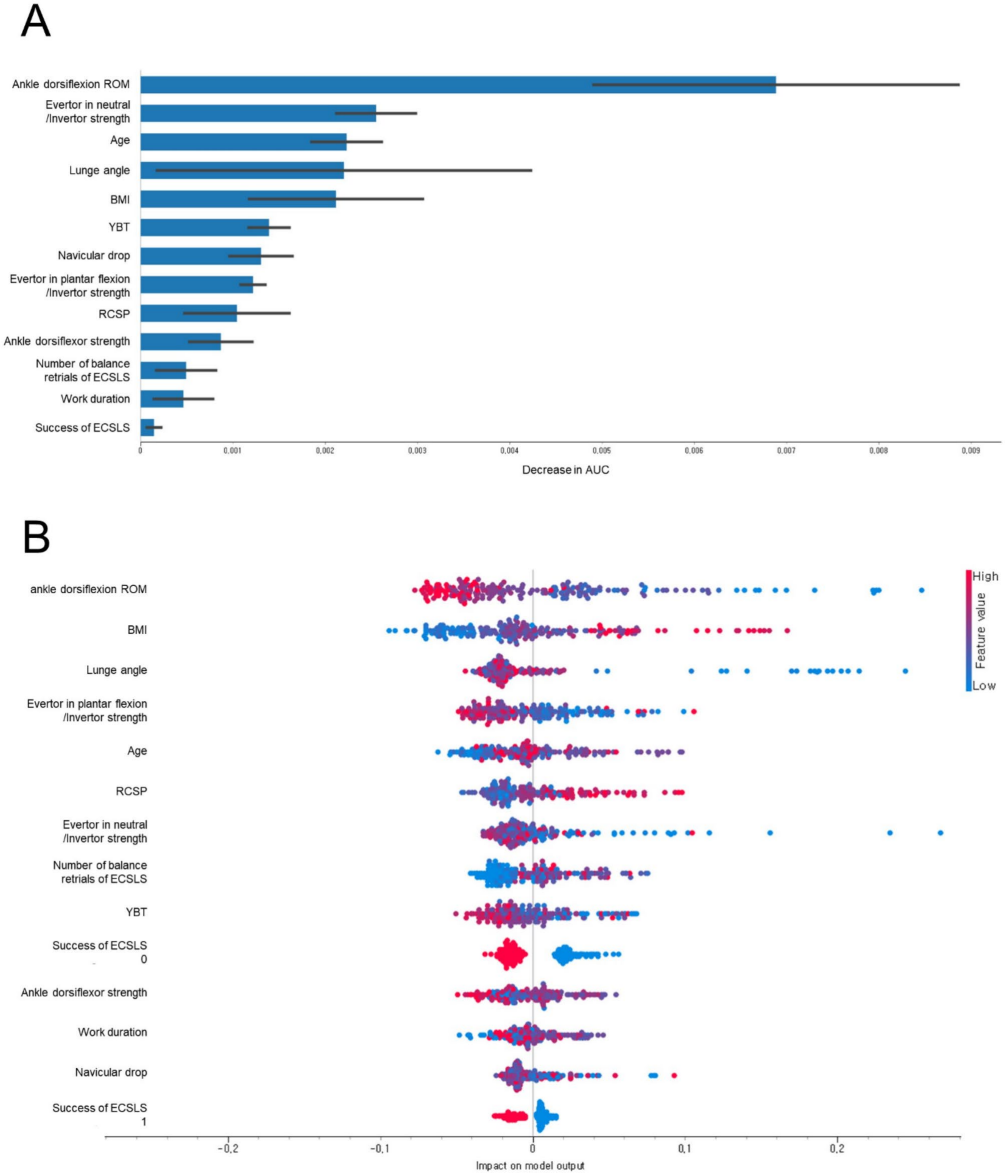
**A**: Feature permutation importance of random forest model in the training set, **B**: Shapley Additive Explanations (SHAP) analyses of random forest model in the training set

Regarding feature permutation importance, the success of the ECSLS, ankle dorsiflexion ROM, RCSP, number of balance retrials of the ECSLS, age, and the strength ratio of the evertor in the neutral position to the invertor position were the most important predictors of CAI in support vector machine model. The ankle dorsiflexion ROM, evertor strength ratio in the neutral position to the inverter, age, lunge angle, BMI, and YBT were the most important predictors of CAI in the random forest model (Fig. 3). For the Shapley Additive Explanations, failure of the ECSLS, low ankle dorsiflexion ROM, a high number of balance retrials of the ECSLS, low strength ratio of the evertor in the neutral position to the invertor, low lunge angle, and high RCSP were the most important predictors of CAI in support vector machine model. Additionally, a low ankle dorsiflexion ROM, high BMI, low lunge angle, low evertor strength ratio in the neutral position to the invertor, old age, and high RCSP were the most important predictors of CAI in the random forest model (Fig. 3).

## Discussion

This study demonstrated that machine learning approaches can effectively classify PDWs with and without CAI using a combination of clinical measurements. The random forest model showed the best performance (AUC = 0.853, good; F1 score = 0.747) among the five machine learning models tested. Multiple machine learning models were employed in this study to enhance the robustness of our findings, as different algorithms provide complementary insights into the classification problem. This approach allowed us to identify consistent predictive factors across different models, increasing confidence in their clinical relevance. The ankle dorsiflexion ROM, evertor strength ratio in the neutral position to the inverter, age, lunge angle, BMI, and YBT were the top predictors of CAI in the random forest model of feature permutation importance. Low ankle dorsiflexion ROM, high BMI, low lunge angle, low evertor strength ratio in the neutral position to the invertor, old age, and high RCSP were the top predictors of CAI in the random forest model with Shapley Additive Explanations. Among the five ML models, the variables entered into three or more models for feature imputation importance were ankle dorsiflexion ROM (five times), age (four times), strength ratio of the evertor in the neutral position to the invertor (three times), RCSP (three times), lunge angle (three times), and BMI (three times). Moreover, among the five ML models, the variables entered into three or more models for the Shapley Additive Explanations were low ankle dorsiflexion ROM (five times), low lunge angle (four times), high BMI (three times), old age (three times), a high number of balance retrials of the ECSLS (three times), and low evertor strength ratio in the neutral position to the invertor (three times). Occupational risk factors for CAI can be difficult to control, manage, or prevent because ankle injuries occur in uncontrolled, unpredictable, and varied outdoor environments during delivery in PDWs [10]. These findings suggest specific targets for clinical intervention in PDWs: (1) ankle dorsiflexion ROM and lunge angle can be improved through joint mobilization techniques and stretching exercises, (2) the evertor-to-invertor strength ratio can be addressed through targeted muscle strengthening programs focusing on the ankle evertors, (3) balance training incorporating visual restriction (eyes-closed) conditions can help address postural control deficits, and (4) BMI management through appropriate exercise and lifestyle modifications should be considered as part of a comprehensive prevention program. These targeted interventions could be implemented in workplace wellness programs or physical therapy settings to prevent and manage CAI in PDWs.

Several studies have reported the performance of risk factor models for ankle sprains and CAI.(33–35) To predict ankle sprains occurring during on-duty physical exercise in firefighters, the lower limb asymmetries of the YBT (AUC = 0.761–0.902; sensitivity: 0.778–1.000; specificity: 0.800–0.889) and weight-bearing lunge test (AUC = 0.844; sensitivity: 0.667; specificity: 0.933) were predictors of ankle sprains [33]. The YBT and single-leg hop test were used to identify adolescent soccer players who experienced lateral ankle sprains. The YBT posteromedial (AUC = 0.78, sensitivity: 0.83, specificity: 0.77) and posterolateral directions (AUC = 0.82, sensitivity: 0.92, specificity: 0.65) and single-leg hop test (AUC = 0.77, sensitivity: 0.67, specificity: 0.94) were predictors of ankle sprains [34]. The AUC value for the ankle inversion discrimination apparatus in the landing test to distinguish between participants with and without CAI for assessing proprioceptive deficits was 0.756 (sensitivity: 0.733; specificity: 0.800) [35]. The present study confirmed that the random forest ML prediction model showed high performance [AUC = 0.853 (good); sensitivity: 1.000, specificity: 0.773, accuracy: 0.887] during the test model classified PDWs with and without CAI. Due to their multifactorial contribution to ankle sprain injuries, impaired balance, postural control, ankle muscle strength, and ankle joint ROM are likely to contribute to ankle instability by reducing the ability of an individual to stabilize the ankle joint against inversion sprains [18, 22]. Thus, classifying CAI using various variables (balance, postural control, ankle muscle strength, ankle joint ROM, and anatomical deformities) may improve the performance of the models presented in this study. The random forest and support vector machine models showed higher performance classification between PDWs with and without CAI than the logistic regression model in our study. The superior performance of these models may be due to their ability to capture complex relationships in the data. However, the specific nature of these relationships would require further investigation with dedicated analyses comparing linear and nonlinear aspects of the associations between CAI and the predictor variables.

For the Shapley Additive Explanations, among the five ML models, the most frequently entered variables were low ankle dorsiflexion ROM (five times) and low lunge angle (four times). Individuals with CAI display less ankle dorsiflexion ROM than healthy controls during running [36] and when landing from a jump [37] due to restrictions in the talocrural joint [27] and distal tibiofibular joint kinematics [38]. Significant differences in lunge angle were confirmed among the three groups with CAI based on the lateral step-down test quality of movement categories (good, moderate, and poor), and there was a negative correlation (*r* = -0.39) between lunge angle and lateral step-down test scores [15]. Due to the nature of delivery work, PDWs are repeatedly exposed to going up and down stairs or getting in and out of cars with parcels and have to run or walk fast to deliver to many destinations in a limited time. If sufficient dorsiflexion does not occur in these motions, ankle instability may occur owing to exposure to a state of relative plantar flexion, which tends to cause ankle sprain. However, it is necessary to determine whether CAI occurs more frequently in PDWs with limited ankle dorsiflexion ROM in future studies because our study design was cross-sectional.

Our study found that high BMI was one of the significant predictors of CAI in PDWs. While Vuurberg et al.‘s meta-analysis showed that patients with lateral ankle sprains had higher BMI compared to healthy controls (with no history of ankle sprains), and those with CAI had higher body weight compared to healthy controls, our study extends these findings by demonstrating that BMI can help distinguish between individuals with CAI and those without CAI (including both individuals with no ankle sprain history and those who have experienced ankle sprains without developing instability, often termed “copers”) [39]. This suggests that BMI may not only be associated with initial ankle sprain risk but may also be an important factor in differentiating between those who develop chronic instability and those who do not follow an ankle sprain. These relationships between body composition measures and ankle stability are particularly relevant in PDWs, who regularly carry heavy parcels, potentially increasing stress on the ankle joint. Regarding age, our findings showed it was a significant predictor, with older PDWs more likely to present with CAI. This finding is particularly relevant to the occupational context of parcel delivery, where workers of all ages must maintain similar work outputs. While previous studies have shown that age itself may not independently predict initial ankle sprains [40, 41], our findings suggest that in PDWs, older age combined with occupational demands may influence the development of CAI. This might be related to age-associated changes in neuromuscular control and strength, which become particularly relevant in physically demanding occupations. This relationship between age and CAI in occupational settings warrants further investigation, particularly considering the physical demands of parcel delivery work.

Numerous balance retrials of the ECSLS and a low strength ratio of the evertor in the neutral position to the invertor were entered thrice in the five ML models. Individuals with CAI have significantly impaired postural stability during ECSLS compared with uninjured controls [18, 42] Postural control deficits in individuals with CAI could be due to motor impairment, somatosensory impairment, or both [22]. During ECSLS, the somatosensory system, altered due to repeated ankle sprains and visual disturbances, is diminished when participants close their eyes [42]. If PDWs carry parcels with both hands and read addresses during delivery, it is easy to impose visual restrictions on the environment under the feet of PDWs. Thus, postural control deficits may have contributed to CAI in PDWs in our classification models. Weak ankle muscle strength likely contributes to ankle instability by decreasing the ability of individuals to stabilize the ankle joint against inversion sprains [18]. Regarding muscle strength, our results present an interesting pattern. While both evertor and invertor strength were significantly lower in the CAI group compared to controls, the evertor-to-invertor strength ratio did not show significant between-group differences in traditional statistical analysis. However, this ratio emerged as an important predictor in our machine learning models. This suggests that while the absolute strength of both muscle groups is reduced in CAI, the relative balance between evertors and invertors (their strength ratio) may have a more complex relationship with CAI than can be detected through traditional statistical comparisons. This finding aligns with biomechanical principles where the relative strength between antagonist muscles is crucial for joint stability [29, 43]. The importance of this ratio in our ML models, despite no significant group differences in direct comparisons, highlights the value of machine learning approaches in identifying complex relationships that might not be apparent through traditional statistical methods.

### Limitations

The present study has some limitations. First, the limited sample size and data imbalance ( CAI = 24.6%; without CAI = 75.4%) may have increased the risk of overfitting. Further studies with larger and wider populations are warranted to improve the predictive efficacy of ML in CAI. Second, while our study used the validated Ankle Instability Instrument for screening CAI, we acknowledge two limitations in our assessment methods. First, incorporating additional patient-reported outcome measures such as the Identification of Functional Ankle Instability, Foot and Ankle Ability Measure, or Cumberland Ankle Instability Tool could have provided more comprehensive assessment of functional limitations and instability symptoms. In addition, while we confirmed the presence of giving way episodes, we could not quantify their frequency due to the yes/no nature of the Ankle Instability Instrument question regarding giving way episodes. Future studies should consider including multiple validated tools that can capture both the presence and frequency of instability symptoms to enhance the robustness of CAI classification. Third, there is a possibility that the best-performing model was not one of the five models selected because of the diversity of ML algorithms. Fourth, we did not measure pathomechanical impairments such as pathologic laxity and arthrokinematic restrictions or sensory-perceptual impairments such as somatosensation and kinesiophobia, which may have contributed to CAI. If pathomechanical and sensory-perceptual impairments were added to our model, the model performance might increase. Fifth, although there are statistical tests to compare different ML algorithms based on a single test dataset, a separate validation study could be needed for verification. Sixth, although our machine learning models showed good predictive performance, these findings should be considered preliminary until validated through prospective studies. Future research should test these predictive algorithms in practice to confirm their clinical utility in identifying PDWs at risk for CAI.

## Conclusions

Our approach of using postural control, ankle ROM, ankle joint muscle strength, and anatomical deformity variables produced clinical ML models to classify PDWs with and without CAI and confirmed good predictive performance in PDWs. For the Shapley Additive Explanations, among the five ML models, the variables entered into three or more models were low ankle dorsiflexion ROM, low lunge angle, high BMI, old age, a high number of balance retrials of the ECSLS, and low strength ratio of the evertor in the neutral position to the invertor. The variables contributing to the model performance regarding feature importance can be considered as clinical guidelines for preventing and managing CAI in PDWs.

## Data Availability

The datasets analyzed during the current study are available from the corresponding author on reasonable request.

## Abbreviations

AUC: Area under the curve
BMI: Body mass index
CAI: Chronic ankle instability
ECSLS: Eyes-closed single-limb stance
ML: Machine learning
PDW: Parcel delivery workers
RCSP: Resting calcaneal stance position
ROM: Range of motion
YBT: Y-balance test
